# Supplementary material for: Nonclinical Evaluation of Single-Mutant E. coli Asparaginases Obtained by Double-Mutant Deconvolution: Improving Toxicological, Immune and Inflammatory Responses
Source: Int J Mol Sci. 2024 May 30;25(11):6008. doi: 10.3390/ijms25116008 (PMC11172649; doi:10.3390/ijms25116008)

SUPPLEMENTARY MATERIAL

Figure S1: Chromatograms of SEC to confirm purity of each ASNase type II, WT and mutants S206C and P40S. SEC was performed in Superdex 200 Increase 10/300 GL column and eluted using 50 mM Tris HCl and 100 mM glycine pH 7.4 with a flow rate of 1 mL/min on 5 column volume.

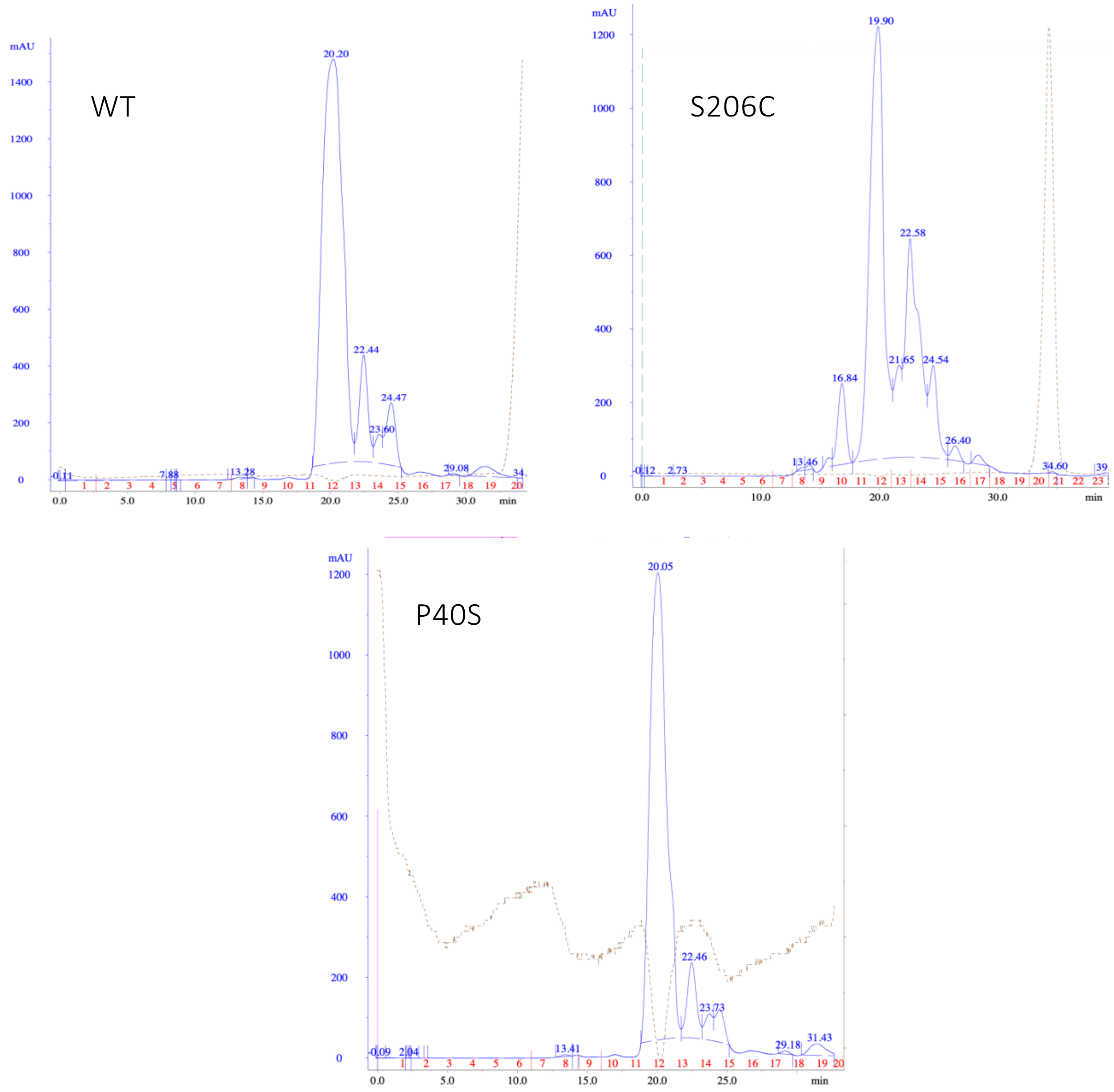

Figure S2: SDS-PAGE gels (14%) to confirm purity of each ASNase type II, WT and mutants S206C and P40S. Elution fraction #12 was used for further tests.

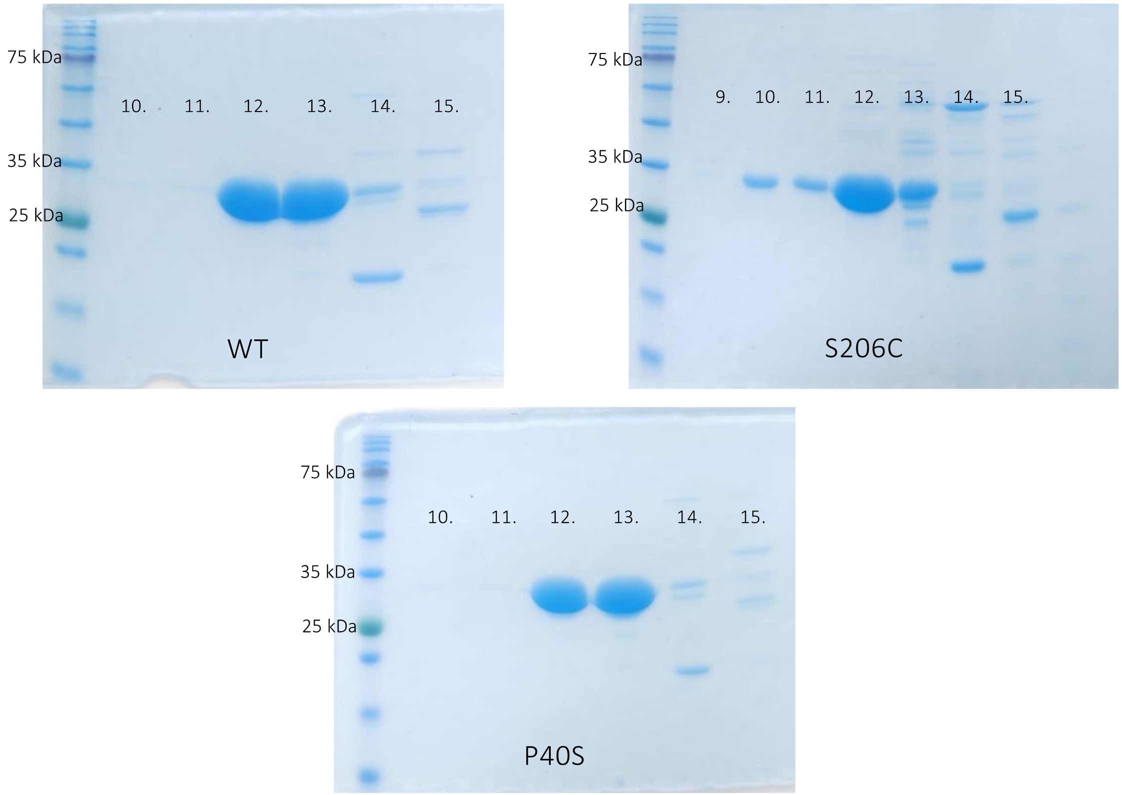

Figure S3: (A) Asparaginase activity of WT, S206C, P40S and P40S/S206C enzymes. The enzyme specific activity is represented as the slope of the linear regression equation of plotted µmole NH_3/min against the milligrams of enzyme (U/mg). n=3. (B) Glutaminase activity of WT, S206C, P40S and P40S/S206C enzymes. The slope value corresponds to the glutaminase specific activity in U/mg. As well as asparaginase activity, µmole of NH_3 released was quantified by Nessler´s reagent, n=3.

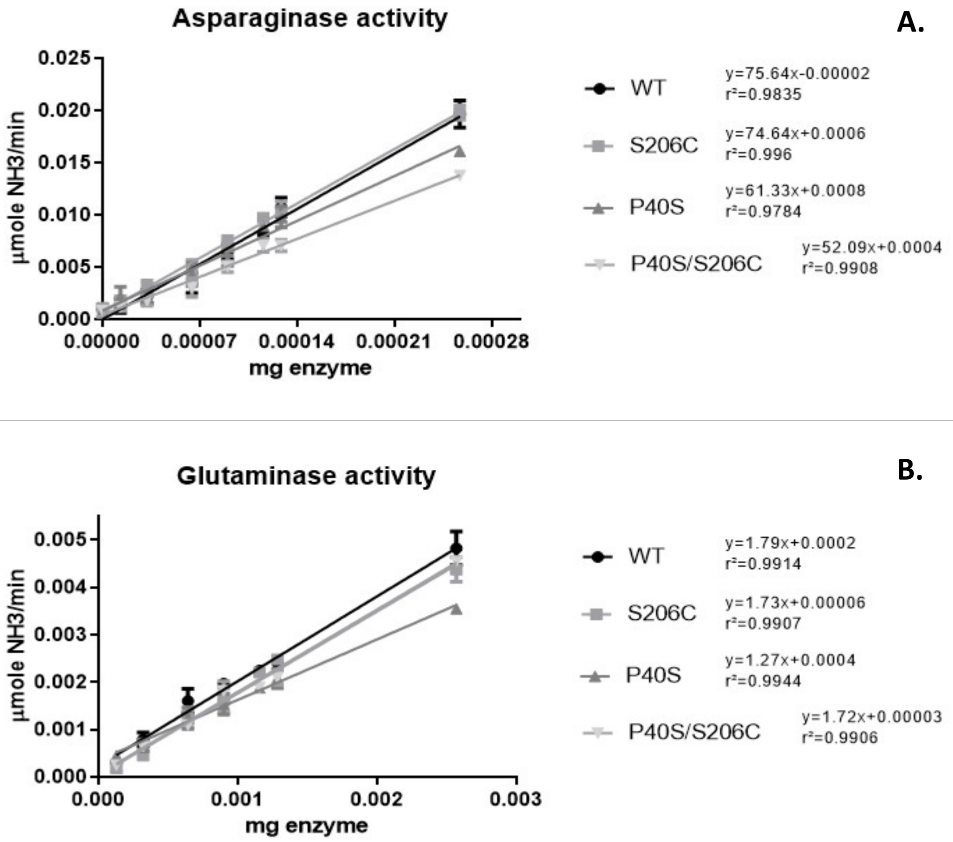

Figure S4: Enzyme stability on human serum (HS). Enzymes were incubated at 37 °C on PBS 1x and on 10% HS up to 96 hours and ASNase activity was measured every 24 hours with Nessler´s reagent. Statistical analyses were significant from 72 hours (A) and 96 hours (B). Standard deviation (SD) is shown by vertical bar, n=2, *: p value <0.05, **: p value <0.005, ***: p value <0.0005.

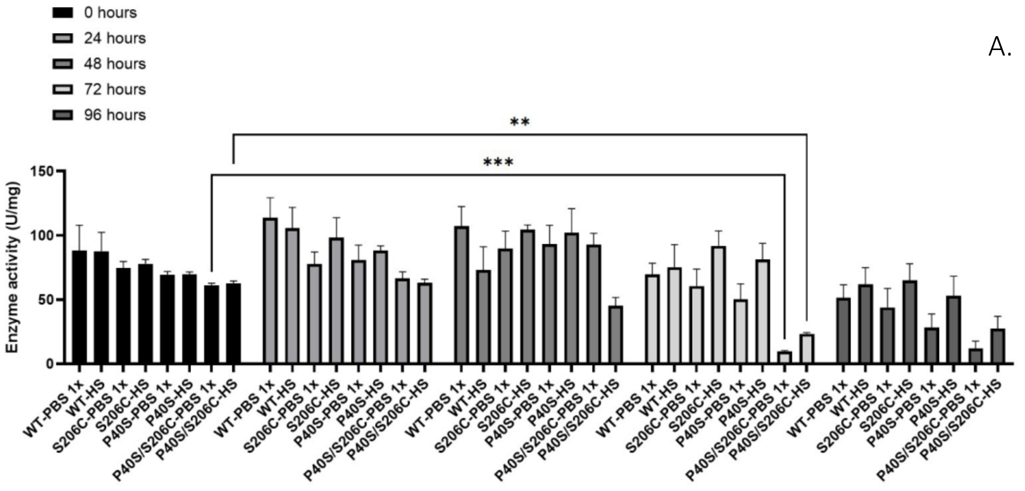

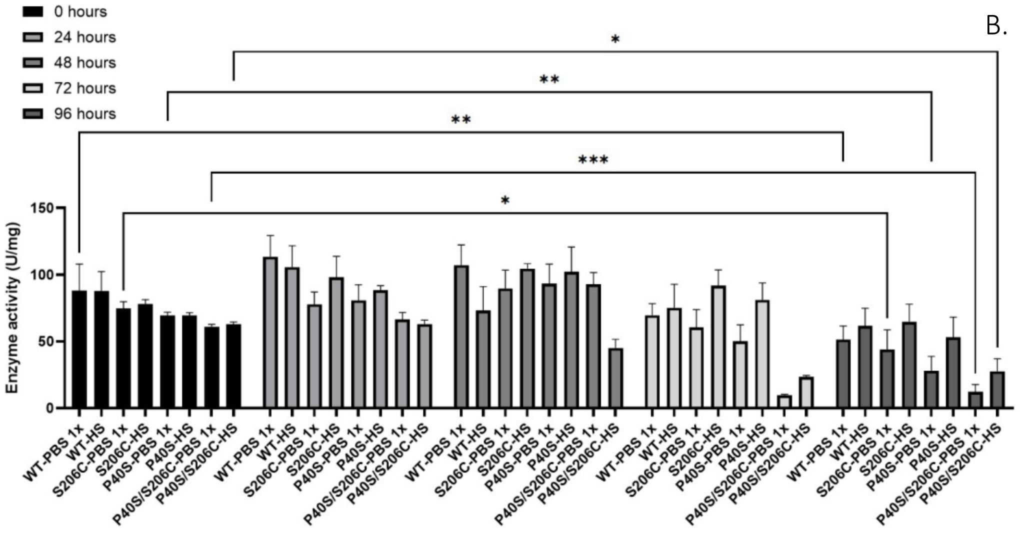

Figure S5: Haematoxylin & Eosin stain of liver, kidney and heart of all enzyme groups. Arrows show microvesicular steatosis on liver, acute tubular necrosis on kidney and mild intercellular edema on heart.

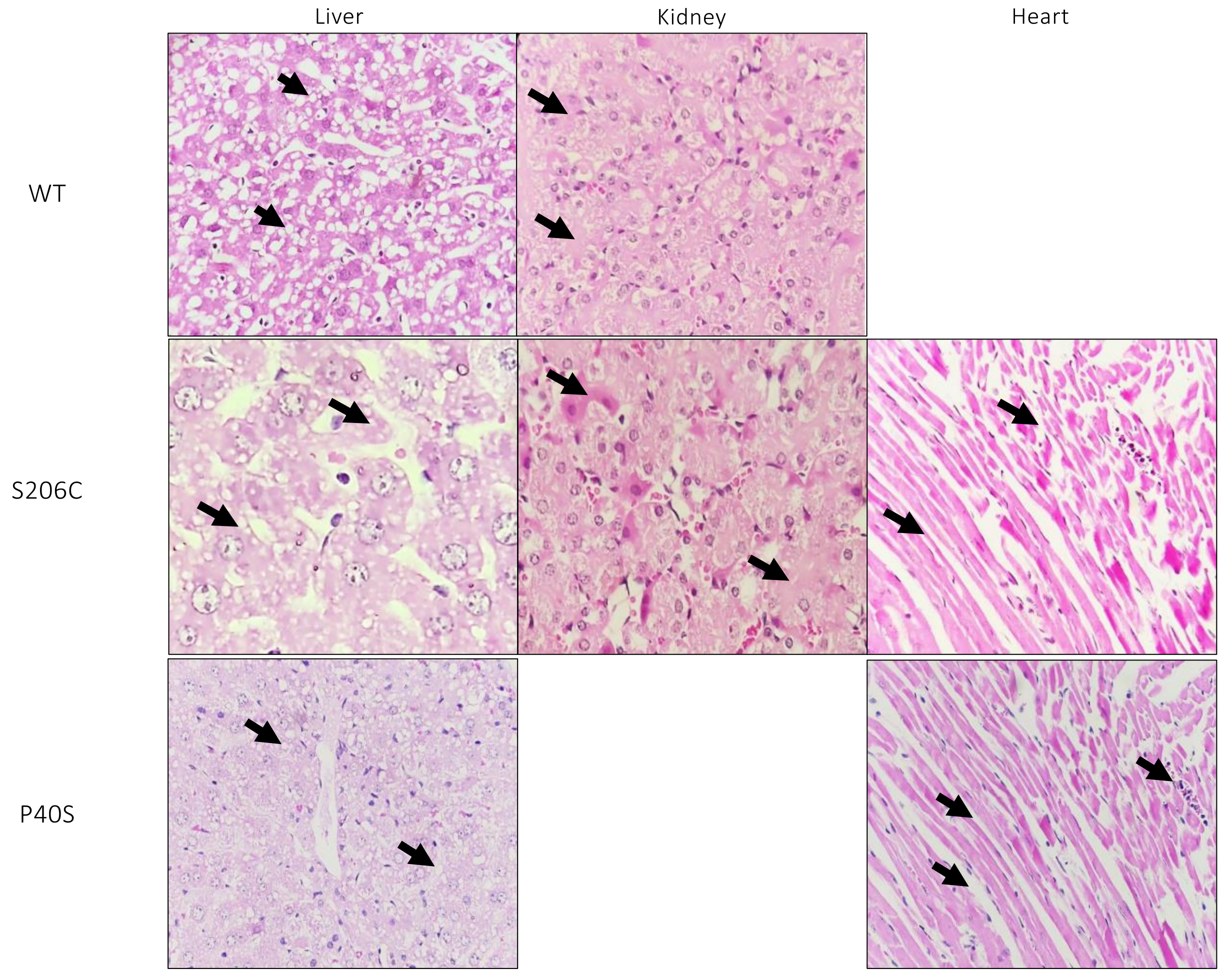

Supplement: Supplementary file 1 [file ijms-25-06008-s001.zip › ijms-2995932-supplementary.pdf]
